# Low-intensity extracorporeal shock wave therapy for pediatric respiratory diseases: current evidence and translational barriers

**DOI:** 10.3389/fped.2026.1876906

**Published:** 2026-10-27

**Authors:** Xingyue Su, Die Liu, Jing Liu, Di Lv, Jianning Guo, Qin Hui, Fang Ye, Yuanmei Chen, Chao Wang, Lijuan Tang, Meihong Xia, Qi Zhang

**Affiliations:** 1 Department of Pediatrics, China-Japan Friendship Hospital (Institute of Clinical Medical Sciences), Beijing, China; 2 Chinese Academy of Medical Sciences and Peking Union Medical College, Beijing, China

**Keywords:** extracorporeal shockwave therapy, respiratory tract diseases, child, ethics research, translational research biomedical

## Abstract

Low-intensity extracorporeal shock wave therapy (Li-ESWT) has demonstrable mechanobiologic activity in nonpulmonary systems, where it has been linked to adenosine triphosphate (ATP) release and downstream signaling, macrophage and fibroblast responses, matrix remodeling, and tissue repair. Whether those effects extend to pediatric respiratory disease is uncertain. Adult clinical studies indicate biologic responsiveness in selected inflammatory or reparative settings, but effect sizes are variable and durability is often undemonstrated. Direct pulmonary evidence comes from bronchial cell cultures, including chronic obstructive pulmonary disease (COPD)-derived fibroblasts, and acute lung injury/acute respiratory distress syndrome models. These show biological responses without establishing a calibrated lung-tissue dose or a pulmonary safety window. The air-filled, acoustically heterogeneous lung presents physical challenges to pressure-wave delivery that nonpulmonary experience cannot resolve: interface reflections, inflation-dependent energy scatter, and uncertain lesion depth collectively mean that energy deposition in lung tissue remains poorly characterized. In children, these physical uncertainties are compounded by ongoing alveolarization, age-dependent chest-wall mechanics, and the absence of pediatric device data. This narrative review integrates cross-organ mechanistic studies, pulmonary cellular and animal data, pediatric respiratory biology, and ethics and device-governance literature to define what lung-directed preclinical work is needed and why pediatric pulmonary trials are not yet justified. Pulmonary dosimetry, injury-threshold characterization, standardized adverse-event monitoring, and adult pulmonary pilot safety data must precede any consideration of pediatric enrollment.

## Introduction

Chronic inflammation, abnormal repair, and progressive remodeling are recurring themes across pediatric respiratory disease—in airway remodeling during recurrent wheeze and asthma, developmental arrest and structural airway abnormalities in bronchopulmonary dysplasia (BPD), and disordered repair following severe infection, mechanical ventilation, or inhalational injury (5, 19–25, 36–40). Low-intensity extracorporeal shock wave therapy (Li-ESWT) attracts interest in this context because nonpulmonary studies describe effects beyond symptom palliation: adenosine triphosphate (ATP) release and downstream kinase activation, cytoskeletal and immune-cell responses, angiogenic signaling, regulation of fibrosis-related molecules, and measurable activity in inflammatory and injury-repair settings (1–18, 41–43).

These observations do not transfer cleanly to the pediatric lung. The lung is air-filled, interface-rich, acoustically heterogeneous, and structurally uneven, so pressure waves behave very differently there than in soft-tissue organs. In children, that problem sits on top of ongoing alveolarization, an immature chest wall, and developmental mechanobiology that has no adult equivalent (19, 24, 25, 28, 44). Airway remodeling, developmental lung injury, and post-injury fibrotic change are the pulmonary settings most closely aligned with the inflammatory and reparative contexts already studied with Li-ESWT. Cystic fibrosis, bronchiolitis obliterans, and pediatric interstitial lung disease may also be relevant, but the published literature is too thin to support disease-specific claims (5, 19–25).

This narrative review synthesizes cross-organ mechanistic studies, pulmonary cellular and animal data, pediatric respiratory biology, and ethics and device-governance literature to define the evidentiary boundary and key translational barriers for Li-ESWT in the pediatric lung; source-level study characteristics and the staged translational pathway are summarized in Supplementary Tables S1–S3.

## Review approach

Literature selection was PubMed-centered and iterative, supplemented by backward reference screening and targeted consultation of pulmonary mechanobiology, pediatric respiratory biology, research ethics, and device-governance literature. The review addresses three linked questions: which biological responses have been demonstrated after shock-wave exposure, how directly these findings apply to the lung, and what developmental and safety considerations affect pediatric applicability. Original experimental studies were used to examine mechanisms and pulmonary responses; systematic reviews were used to contextualize nonpulmonary clinical findings where available.

Pulmonary cell and animal studies were discussed separately from nonpulmonary experiments and clinical studies. Mechanobiology studies without shock-wave exposure were included to explain lung repair and injury pathways, not as evidence of a Li-ESWT treatment effect. Pediatric developmental, ethical, and device studies provided the clinical context. The approach was narrative rather than protocol-exhaustive; no formal risk-of-bias grading or quantitative pooling is presented. The search approach and selected study characteristics are described further in the Supplementary Methods and Tables S1–S3.

## Pediatric respiratory context

### Airway remodeling and developmental injury

The long-term burden of pediatric respiratory disease does not come from recurrent inflammation alone. It also reflects a lung that fails to return to a normal developmental trajectory after injury. In childhood asthma, remodeling includes basement membrane thickening, increased smooth muscle mass, epithelial injury, and fibrosis-related mediators. Systematic review data indicate that structural change may already be present in early childhood, though its temporal relationship to inflammation and its causal role in functional decline remain incompletely resolved (20). Bronchoscopic and pathological studies document early reticular basement membrane thickening, associated airway inflammatory and structural changes, and correlations between that thickening and clinically relevant disease features (45–47). Analyses of fatal pediatric and adolescent asthma likewise support a remodeled airway phenotype (48), and more recent work linking fibrotic mediators to functional measures reinforces the view that long-term outcomes are shaped by structural change as well as by symptom burden (22).

Phenotypic heterogeneity is consequential here. Many pediatric cases remain predominantly inflammatory and treatment-responsive. Others show a more remodeling-dominant or steroid-refractory profile, and it is within this latter group that stromal or matrix-directed approaches carry greater mechanistic plausibility (20, 22, 45–49). Airway compression, epithelial deformation, and mesenchymal mechanosensing may contribute to remodeling without supplanting canonical inflammatory pathways, though the mechanistic overlap is narrow (49). Within asthma specifically, type 2-high (T2-high) disease—driven by eosinophilic and type 2 cytokine activity—tends to be more corticosteroid-responsive and represents a weaker theoretical fit for mechanical intervention. Type 2-low (T2-low) or steroid-resistant subtypes show greater matrix change and remodeling, making them a more plausible mechanobiologic target, but these subgroups are less prevalent in pediatric populations and their mechanical drivers are incompletely characterized (20, 22). Any Li-ESWT model of pediatric asthma would therefore need to specify the inflammatory-remodeling endotype rather than assume a single transferable biology.

In BPD, the central issue is interrupted development. Preterm birth, oxygen exposure, and mechanical ventilation can affect airway maturation, alveolar growth, and pulmonary vascular development (19, 24). Long-term outcome data emphasize persistent respiratory morbidity and heterogeneous lung-function trajectories, particularly in severe disease (50). BPD is therefore best understood in a life-course context, even when supplemental oxygen or ventilatory support can later be withdrawn.

Respiratory-support-based severity and long-term structural recovery describe different aspects of BPD. Lower oxygen or support requirements do not establish normal alveolar development or exclude later airway dysfunction. Conversely, greater support requirements indicate a higher clinical burden without identifying a uniform degree of fibrosis or a stronger expected response to a matrix-directed intervention (19, 24, 50). Preclinical models should distinguish developmental stage, prior oxygen and ventilation exposure, airway and alveolar abnormalities, and vascular involvement rather than infer mechanobiologic treatment suitability from a mild, moderate, or severe label alone.

### Mechanobiology

Pulmonary research now treats the mechanical microenvironment as part of the disease process, not just its aftermath. Extracellular matrix stiffening, abnormal stretch, epithelial-mesenchymal crosstalk, and fibroblast activation each contribute to pathologic lung mechanics (23, 25, 40). Lung-specific studies have given this overlap more concrete form: yes-associated protein/transcriptional coactivator with postsynaptic density protein, Drosophila disc large tumor suppressor, and zonula occludens-binding motif (YAP/TAZ) signaling, integrin *α*v*β*6-mediated activation of latent transforming growth factor-beta (TGF-*β*), and epithelial-mesenchymal feedback loops have been implicated in fibroblast activation, fibrotic amplification, alveolar repair and development, and macrophage-driven pulmonary fibrosis programs (51–57, 65). Shared pathway biology helps explain why Li-ESWT keeps reappearing in pediatric respiratory discussions, but it is not transferable evidence. Mechanistic overlap can justify experiments; it cannot by itself justify clinical translation (1, 23, 41–43, 51–57, 65). At most, it suggests that preclinical models should test whether Li-ESWT changes inflammatory amplitude after airway or alveolar injury, alters the sequence from epithelial damage to mesenchymal activation, or shifts remodeling-associated markers beyond the short-term inflammatory readouts used in nonpulmonary studies (3–5, 51–58).

### Limits of extrapolation

Pediatric respiratory medicine compounds this difficulty: the outcomes that matter most emerge slowly and resist direct measurement. Lung tissue is rarely available for mechanistic study, long-term functional change unfolds over years, and biomarkers of pulmonary health are still in early development (21). For Li-ESWT, the first issue is not effect size. It is whether there is any preclinical basis strong enough to justify studying the therapy responsibly in pediatric respiratory disease at all (9, 10, 21, 31, 34, 35).

## Cross-organ mechanisms

### Parameters

Extracorporeal shock wave therapy (ESWT) encompasses focused and radial or defocused forms that differ in wave source, energy distribution, penetration depth, and focal control, making them non-interchangeable technologies (41–43). For Li-ESWT, the practical question is which combination of wave source, energy flux density, pulse number, and target depth can produce a biologic response without causing injury. Even in tendon, wound, and urologic applications, that window appears tissue-specific, which makes direct parameter transfer to lung implausible (1, 2, 9, 11, 12, 41–43).

For this review, Li-ESWT refers operationally to regenerative, non-lithotripsy protocols at low energy settings—commonly below roughly 0.2 millijoules per square millimeter (mJ/mm^2^) in the urologic literature, though no universal cutoff is accepted across devices or indications (1, 41, 42). Dose-response relationships remain poorly defined even in mature nonpulmonary indications, and this uncertainty carries over directly into pulmonary safety interpretation (9–16). Therapeutic protocols are typically described by low energy flux density and low-Hertz (Hz) repetitive delivery. The Tong mouse study, by contrast, is a pressure-wave lung-injury model, not a therapeutic Li-ESWT protocol: it used a simulated blast system in which rupture of a 1.4 mm aluminum film generated peak overpressure around 337 pounds per square inch (PSI) (approximately 2.3 megapascals) (28). The numbers are not directly comparable to therapeutic ESWT reporting, but the underlying point is clear—lung tissue is vulnerable to rapidly transmitted pressure waves, and establishing an injury boundary must precede any efficacy claim.

### Adenosine triphosphate-purinergic and extracellular signal-regulated kinase signaling

The most direct experimental sequence linking mechanical stimulation to downstream biology runs through ATP release. *In vitro* wound-healing models show that shock waves promote ATP efflux, activate extracellular signal-regulated kinase 1/2 (ERK1/2) and p38 mitogen-activated protein kinase (MAPK), and modulate proliferation, migration, and repair responses (2). Purinergic receptor P2X ligand-gated ion channel 7 (P2X7) receptor participation in shock-wave-induced osteogenic differentiation has been demonstrated in human mesenchymal stem cells (6). Together these studies show that mechanical stimulation can connect to pathways involved in stress responses, repair, and remodeling, but all of this evidence comes from outside the lung (2, 6). It supports mechanistic plausibility, not a lung-specific pathway. In pulmonary settings, ATP release could feed epithelial repair programs, macrophage inflammatory signaling, or both, depending on local cell state, receptor landscape, and injury context. The release route itself remains undefined in Li-ESWT studies, making spatial and temporal distribution of any pulmonary ATP signal difficult to predict (1, 2, 6, 26, 27).

### Mechanobiology as context

Piezo1 is a calcium-permeable, nonselective cation channel. In human dental-pulp mesenchymal stem cells, the chemical activator Yoda1 induced entry of extracellular Ca^2^⁺ into the cytosol and increased intracellular calcium (7). ATP was released from the cells into the culture medium, and ATP degradation or broad P2 receptor inhibition suppressed the associated migration response. Thus, inward calcium conduction and outward ATP release are distinct events; the study did not identify the ATP-release route, establish calcium entry as the obligatory cause of ATP release, or determine the downstream P2 receptor subtype (7). This was a chemical-activation experiment, not a shock-wave experiment.

Macrophage studies also link Piezo1-dependent calcium entry to stiffness sensing and inflammatory responses (8). These observations support a candidate mechanosensing pathway, not a demonstrated pulmonary Li-ESWT mechanism. Nuclear mechanotransduction studies provide additional context for transcriptional responses that can outlast brief ionic events (59, 60).

YAP/TAZ are mechanoresponsive drivers of fibroblast activation and contractile programming (51, 52), while epithelial YAP/TAZ activity contributes to alveolar regeneration and morphogenesis (55–57). Integrin *α*v*β*6 provides a separate route for activation of latent TGF-*β* and has been implicated in pulmonary inflammation and fibrosis (53, 54). None of the three pulmonary shock-wave studies discussed here measured YAP/TAZ or tested *α*v*β*6-mediated latent TGF-*β* activation (26, 27, 29). Limited TGF-*β*-related response data do exist: Lin et al. measured lower lung TGF-*β*, phosphorylated Smad3, and collagen deposition after treatment, and Di Stefano et al. measured cell-dependent changes in TGF-*β*1 and procollagen-I in bronchial fibroblast cultures (26, 29). These observations do not establish an upstream shock-wave mechanotransduction pathway; altered tissue injury can itself change TGF-*β* and matrix-related readouts. Cell-specific pathway experiments would be needed to distinguish epithelial repair from persistent profibrotic fibroblast activation.

### Immune and fibroblast responses

In wound and fibrosis-related studies, the most transferable theme is immune-matrix interaction. Low-intensity shock waves can improve macrophage functions associated with chronic wound healing (3), alter fibrosis-related molecules (4, 58), and modulate fibroblast activation through a transient proinflammatory milieu (5). Across these systems, Li-ESWT seems to affect inflammatory tone, stromal activation, and repair timing rather than acting through one dominant pathway. Whether that pattern holds in the lung remains a hypothesis. The practical value for pulmonary work is the biomarker set it suggests: macrophage phenotypes, epithelial injury-repair markers, fibroblast activation states, YAP/TAZ-dependent contractile programs, and *α*v*β*6-TGF-*β* signaling (3–5, 51–58).

### Pulmonary disease mapping

Airway remodeling offers the clearest mapping between cross-organ mechanobiology and pulmonary disease. In childhood asthma and recurrent wheeze, the relevant issue is whether Li-ESWT can alter epithelial stress, mesenchymal activation, and matrix remodeling in the airway wall (20, 22, 45–49). The cross-organ data on ATP-purinergic signaling, macrophage-fibroblast crosstalk, and stromal activation matter mainly because they point to airway-specific readouts: epithelial injury markers, fibroblast activation states, basement-membrane and matrix-associated mediators, and function-linked remodeling signals (2–8, 22, 45–49, 58).

Developmental lung injury poses a different problem. In BPD and related preterm lung disease, inflammation, interrupted alveolarization, and abnormal repair occur while the organ is still forming (19, 24, 25, 50, 57). What matters here is whether externally delivered mechanical signals disturb injury-repair sequencing further or move it back toward normal development. Answering that requires direct measurement of YAP/TAZ biology, epithelial-mesenchymal crosstalk, and preterm inflammatory vulnerability in the same model (19, 24, 25, 50, 55–57, 61).

The least mismatched setting in the current literature is post-injury repair after severe lung damage. Acute lung injury (ALI) and acute respiratory distress syndrome (ARDS) models already center on epithelial injury, inflammatory cascades, pyroptosis-related pathways, and alveolar recovery (26, 27, 55, 56), and cross-organ immune and fibroblast data justify asking whether Li-ESWT changes macrophage phenotypes, epithelial restitution, fibroblast activation, and restoration of alveolar architecture. Even here, the relevant endpoint is mechanistic pulmonary response, not pediatric treatment readiness (3–5, 26, 27, 55, 56, 58).

### Device heterogeneity

For most nonpulmonary indications, device differences primarily affect effect size and cross-study comparability. In lung translation, they determine whether a safety window can be meaningfully defined at all. Focused and radial or defocused devices differ in focal control, energy distribution, and likely tissue-penetration patterns (41–43), and in the lung these uncertainties are compounded by chest-wall thickness, air-liquid interfaces, lesion depth, and the extent of unavoidable normal-lung exposure (26–28, 44). Pediatric anatomy sharpens the problem further: adult-oriented focal geometry may overshoot a thin neonatal or infant chest wall, while radial delivery eliminates the deep focal zone only by surrendering spatial control. Pulmonary analysis that does not explicitly separate wave-source characteristics from parameter uncertainty risks importing feasibility assumptions from an organ system where they do not belong (26–28, 41–43).

## Cross-organ clinical signals

Cross-organ clinical evidence shows biologic responsiveness in several nonpulmonary systems, but efficacy is mixed and certainty is often modest. The Cochrane review of erectile dysfunction judged much of the evidence to be low or very low certainty with limited long-term confidence (9). Peyronie's disease studies show more consistent pain improvement than structural change (13). Musculoskeletal evidence is heterogeneous across disorders and endpoints (14–16). Chronic wound data support adjunctive benefit rather than replacement of standard care (10). Neurorehabilitation evidence is strongest for spasticity and does not support claims of neural regeneration (17, 18). Table 1 summarizes these cross-organ signals.

**Table 1:** Cross-organ signals.

System	Main signal	Repair implication	Respiratory implication	References
Erectile dysfunction	Short-term functional gain; low certainty	Human responsiveness without durable structural proof	Shows biologic activity, not pulmonary transportability	(9, 11, 12)
Peyronie's disease	Pain signal stronger than structural change	Symptoms may diverge from remodeling	Warns against equating symptom relief with tissue reversal	(13)
Tendon and bone-tendon healing	Cell proliferation, collagen synthesis, delayed-healing responses	Supports repair sequencing and stromal remodeling	Relevant to airway-wall and postinjury repair concepts only indirectly	(14, 15)
Osteoarthritis and musculoskeletal pain	Heterogeneous effects across endpoints	Tissue context and protocol drive outcome	Reinforces the need for lung-specific parameters	(16)
Chronic wounds and diabetic foot ulcers	Adjunctive benefit in impaired healing	Suggests a shift from chronic inflammation toward repair	Most relevant to failed repair, not direct lung use	(3, 10)
Scar and fibrosis-related fibroblast models	Altered fibrosis-related molecules and fibroblast activation	Supports stromal and matrix markers	Points toward fibroblast and matrix markers in lung studies	(4, 5, 58)
Neurorehabilitation and spasticity	Functional signal exceeds regeneration claims	Visible benefit can outpace mechanistic certainty	Cautions against overclaiming pulmonary regeneration	(17, 18)

The distinction between symptomatic improvement and structural repair is particularly relevant to respiratory research. Nonpulmonary studies suggest questions about inflammatory resolution, epithelial repair, and stromal remodeling that can be examined in lung-relevant models. Their clinical outcomes do not measure pulmonary delivery or lung-specific safety.

The relative evidentiary strength of these data strata and the main remaining translational gaps are summarized in Table 2.

**Table 2:** Evidence hierarchy.

Evidence stratum	Representative evidence	Supports	Translational gap	References
Nonpulmonary mechanistic experiments	Wound-healing, stem-cell, macrophage, and fibroblast models	ATP-linked signaling, immune shifts, stromal activation, repair sequencing	Lung efficacy, pulmonary dosing, pediatric safety	(1–8, 58–60)
Pulmonary cellular and animal studies	Bronchial epithelial cell line and COPD-derived fibroblasts; ALI/ARDS models	Pulmonary responsiveness and candidate lung markers	A treatment window, dosimetry, pediatric readiness	(26, 27, 29)
Pulmonary injury and feasibility evidence	Pressure-wave lung injury; blast-lung and contraindication literature	Lung vulnerability and need for feasibility boundaries	A low-intensity pulmonary safety window	(28, 30, 44)
Nonpulmonary clinical syntheses	Erectile dysfunction, chronic wounds, musculoskeletal disorders, spasticity	Human responsiveness and endpoint caution	Pulmonary translation or pediatric pulmonary benefit	(9–18)
Pediatric respiratory context	Asthma remodeling, BPD, lung development, mechanobiology	Why inflammation, repair, and remodeling markers matter	Li-ESWT efficacy in the lung	(5, 19–25, 45–57, 61)
Pediatric ethics and governance	Pediatric ethics, assent, device governance, sedation	Why preclinical and adult bridge work must come first	Justification for pediatric pulmonary trials	(31–35, 62)

## Pulmonary evidence

### Bronchial epithelial cells and COPD-derived fibroblasts

Di Stefano et al. exposed the 16HBE bronchial epithelial cell line and primary bronchial fibroblasts from three COPD donors and three smoking controls to focused piezoelectric shock waves in culture (29). The protocol used a nominal energy flux density of 0.3 mJ/mm^2^, 500 pulses, and 4 Hz. Although the abstract describes low-energy treatment, the Methods label this setting medium-high energy; it exceeds the approximate low-energy range used operationally in this review. The experiment is therefore informative pulmonary cell-response evidence, not an established Li-ESWT lung-safety protocol.

Proliferation-related indices increased after exposure. TGF-*β*1 protein rose at 72 h in control-smoker fibroblasts, without a corresponding significant increase in COPD fibroblasts; procollagen-I increased early in COPD fibroblasts and declined later (29). The authors did not exclude a modest profibrotic effect. These cell- and time-dependent results support pulmonary stromal responsiveness, not uniform antifibrotic action. No intact lung was treated, and neither culture viability nor proliferation establishes safe transthoracic delivery. COPD-derived fibroblasts and an epithelial cell line also do not reproduce the developmental biology of asthma or BPD (19, 24, 29, 50).

### Acute lung injury and acute respiratory distress syndrome (ALI/ARDS) models

Lin et al. treated rats after hyperoxia-induced ARDS using focused shock waves at a nominal 0.15 mJ/mm^2^, with 200 impulses at 6 Hz per lung field in a single session (26). The study included shock-wave-only, mitochondrial-treatment, and combined-treatment groups. Lung injury and fibrosis-related readouts improved, including TGF-*β*, phosphorylated Smad3, and collagen deposition; the combination produced greater improvements than either intervention alone. These tissue-level responses do not identify a fibroblast-specific mechanism, and the study did not include a healthy-lung shock-wave-only safety group.

Guo et al. reported chest treatment at 0.09 mJ/mm^2^, 200 impulses, and 10 Hz in an LPS-induced mouse lung-injury model (27). The study linked improved injury readouts with mitochondrial and pyroptosis-related changes. Its healthy shock-wave-exposed group nevertheless showed minor alveolar atrophy, occasional alveolar fusion, and scant perivascular fibrosis and inflammatory cells, without overt hemorrhage. This comparator provides limited short-term safety information rather than evidence of an injury-free exposure. The separate AT2 culture protocol used 1.5 bar, 500 impulses, and 1 Hz; it should not be treated as equivalent to the animal protocol (27).

These experiments concern acute injury rather than chronic airway remodeling or developmental interruption. Neither reports measured *in-situ* parenchymal dose or tests a YAP/TAZ-dependent response (26, 27). Supplementary Table S2 summarizes the exposure settings and findings. No clinical Li-ESWT study in adult asthma, interstitial lung disease, or pulmonary fibrosis was identified in the literature reviewed here.

## Pulmonary barriers

### Air-filled lung physics

Pulmonary translation is constrained first by physics and anatomy. Shock-wave propagation across air-liquid interfaces differs fundamentally from propagation through solid tissue, making energy transfer across the chest wall, pleura, and parenchyma likely to be heterogeneous and difficult to predict. Experimental lung-injury studies and blast-lung literature both document that rapidly transmitted pressure phenomena can injure air-containing tissue, and device studies indicate that cavitation and interface effects may further amplify local stress (28, 30, 43). The operative question is therefore not whether shock waves can reach the lung in a general sense, but whether a device- and parameter-specific window can be defined in which biologic signaling is achieved without causing injury—a question that cannot be answered by extrapolating from tendon or musculoskeletal protocols (26–28, 30, 44). A concise comparison of device types and extrapolation limits is provided in Supplementary Table S1.

### Dosimetry

The cited pulmonary experiments report nominal device settings and biological responses, not a calibrated spatial distribution of pressure or energy within aerated lung tissue (26, 27, 29). Directly exposing cells in a liquid-filled tube bypasses the chest wall, pleura, and air-containing parenchyma. Lung-tissue responses after rodent chest treatment support biological activity under the tested conditions, but do not quantify the local mechanical exposure or distinguish direct parenchymal effects from secondary changes in injury and inflammation. The existing studies therefore do not demonstrate a lung-tissue dose that is both biologically effective and established as safe.

Device-reported energy flux density and pulse counts cannot be equated with the dose received by a particular lung region. Coupling, thoracic geometry, focal depth, inflation state, and air-tissue interfaces affect transmission; a bar setting in cell experiments is also not interchangeable with energy flux density in animal treatment. The mild abnormalities in healthy exposed mice further caution against interpreting improvement in an injured lung as proof of safety (27). Spatial exposure measurements linked to epithelial and vascular injury, as well as delayed remodeling, would make the relationship between dose and response interpretable. Regional heterogeneity is already familiar in pulmonary drug delivery, although aerosol deposition and shock-wave exposure involve different physical processes (63, 64).

## Pediatric barriers

### Developmental biology

Developmental stage matters more than body size alone. Preterm infants and term neonates have highly compliant chest walls, immature alveolar structures, limited ability to cooperate, and a high likelihood of requiring sedation or anesthesia for any repeatable device-based study (19, 24, 25, 62). As children grow, procedural feasibility improves, but long-term structural consequences remain difficult to infer from short-term safety observations (21, 24, 25). Any future pediatric discussion must therefore specify disease setting, age range, developmental stage, and risk-benefit assumptions instead of treating childhood as a single translational category. An age-stratified framework is summarized in Figure 1.

**Figure 1:**
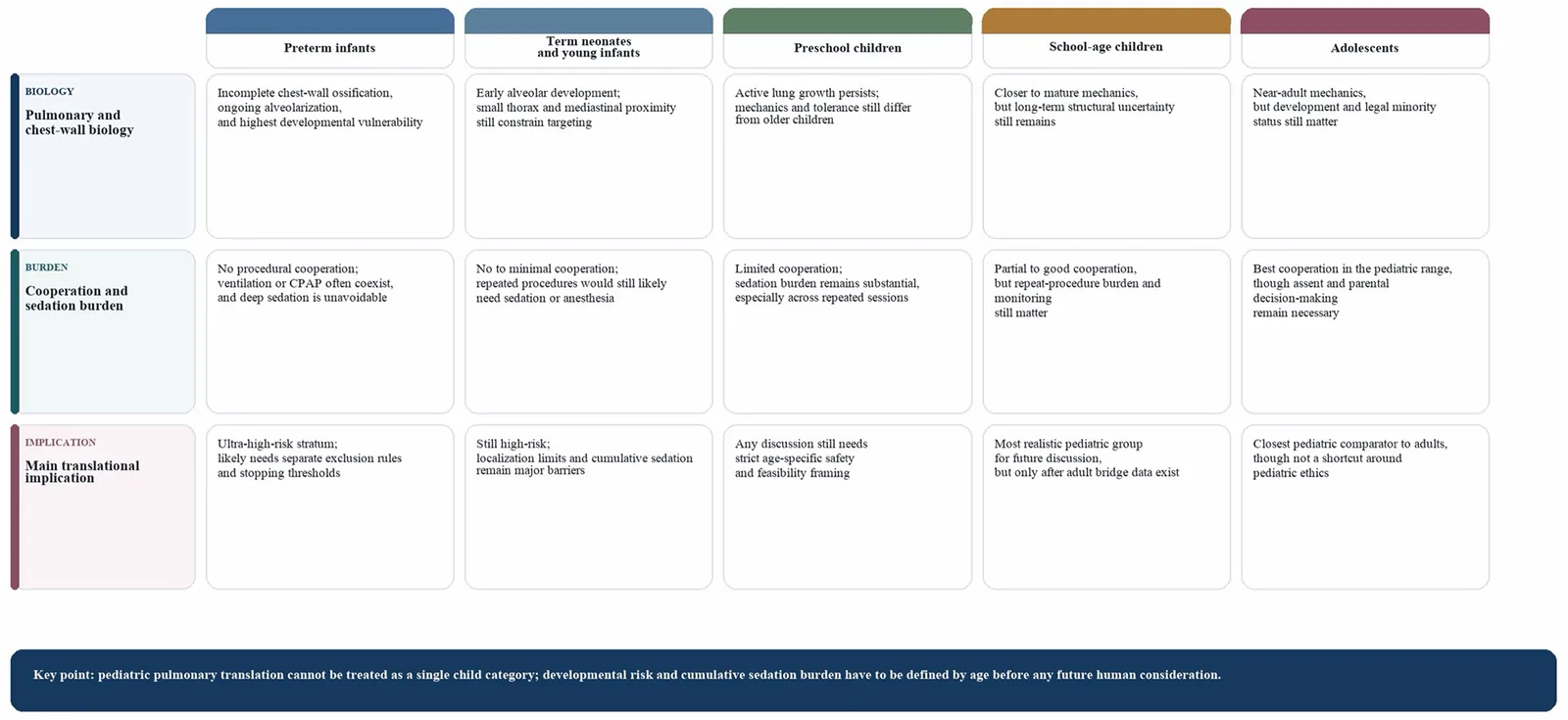
Age-stratified translational risk framework for pediatric pulmonary Li-ESWT. Age-stratified translational risk framework for pediatric pulmonary Li-ESWT. The framework contrasts developmental stage, chest-wall and airway mechanics, need for sedation, and uncertainty about long-term pulmonary consequences across preterm infants, term neonates, infants, preschool children, and school-age children and adolescents. The figure is a heuristic risk map rather than a quantitative scoring system and is meant to highlight why the youngest patients carry the greatest uncertainty. Supporting device-heterogeneity considerations are summarized in Supplementary Table S1.

The youngest patients, particularly preterm infants at risk of BPD, present the greatest developmental uncertainty. Continuous positive airway pressure (CPAP) or mechanical ventilation changes the mechanical conditions of exposure, while preterm immune development may affect mechanically induced inflammatory responses (19, 24, 50, 61). YAP/TAZ activity during alveolar morphogenesis also raises the possibility that external pressure-wave exposure could disturb normal growth programs (57). If this developmental setting is reconsidered, exclusion criteria, monitoring thresholds, and stopping rules would require separate age-specific justification. These interacting factors are relevant to developmental injury models; Table 3 compares BPD with other preclinical disease settings rather than ranking clinical BPD severity.

**Table 3:** Preclinical prioritization.

Disease setting	Pathologic fit	Endpoints	Pulmonary risk	Pediatric complexity	Relative preclinical tractability	Main limitation	References
Asthma airway remodeling	Stronger	Moderate	Uncharacterized	Higher	Intermediate	Readouts remain indirect and invasive	(20, 22, 45–49)
Developmental lung injury or BPD	Intermediate	Lower-moderate	Uncharacterized	Highest	Lower	Ventilation, alveolarization, and immune immaturity raise uncertainty	(19, 24, 25, 50, 57, 61)
Post-injury repair after severe lung injury	Stronger	Moderate-higher	Uncharacterized	Moderate-higher	Relatively higher	Closest to ALI or ARDS biology, but still preclinical only	(26, 27, 50, 55, 56)
Fibrotic lung remodeling or pediatric interstitial lung disease-like trajectories	Intermediate	Lower	Uncharacterized	Highest	Lower	Mechanobiologic relevance exists, but direct Li-ESWT evidence is minimal	(23, 40, 51–54)

This table offers qualitative considerations for preclinical model selection, not a quantitative score or a clinical risk ranking. Pulmonary Li-ESWT risk has not been compared experimentally across these settings. The relative assessments of pathologic fit, endpoint measurability, pediatric complexity, and tractability are author judgments rather than validated comparisons.

Table 3 compares preclinical disease models in terms of pathologic fit, endpoint measurability, pulmonary safety uncertainty, and developmental complexity. Post-injury repair and airway-remodeling models are more directly connected to the available experiments than BPD or heterogeneous fibrotic phenotypes (5, 19–28).

### Endpoints

Endpoint selection is a major obstacle because the outcomes most relevant to pediatric lung health emerge slowly and resist direct measurement. Pediatric pulmonary biomarker work and childhood asthma remodeling studies indicate that reliable noninvasive measures of long-term structural change are still under development (20, 21). Candidate endpoints include exhaled or blood inflammatory markers, oscillometry or infant pulmonary function testing, imaging-based airway or ventilation measures, exacerbation burden, and technology dependence in severe BPD, but none has been validated as a Li-ESWT response marker (20–22, 50). Even if early studies show a signal, the next challenge will be demonstrating that any benefit is durable, developmentally appropriate, and not offset by local or procedural risk. Supplementary Table S2 distinguishes endpoints that have actually been studied from those that are still inferred.

### Ethics

Children present a higher ethical threshold because decision-making capacity is limited, risk bearing is less discretionary, and consequences may extend across growth and development. Pediatric ethics and assent literature emphasizes risk-benefit justification, developmentally appropriate assent, and transparent reporting; pediatric device literature similarly emphasizes the evidence needed to assess device-related uncertainty (31–34, 66). For pulmonary Li-ESWT, both the prospect of direct benefit and the magnitude of risk remain poorly characterized. Table 4 summarizes these ethical considerations. Figure 2 summarizes the relationships between the experimental evidence and unresolved pulmonary and pediatric questions.

**Table 4:** Ethical framework.

Dimension	Status	Ethics question	Judgment	References
Scientific premise	Limited but real	Strong enough for human study?	Supports more preclinical work, not pediatric research	(5, 19–29, 51–57)
Risk category	Poorly bounded	Are pulmonary and sedation risks defined?	More consistent with above-minimal risk	(28, 30–35, 44, 62)
Prospect of direct benefit	Not established	Can direct benefit be credibly claimed?	No	(9, 10, 26–28, 31, 34)
Monitoring plan	Inadequate	Are adverse-event (AE) monitoring and follow-up standardized?	No unified framework yet	(9, 10, 21, 26–28, 32, 33, 35, 62)
Stopping rules	Absent	What should trigger pausing or termination?	Must be defined in adult and preclinical stages first	(26–28, 31, 34, 35)
Device evidence maturity	Sparse	Is the pediatric device evidence base mature enough?	No	(32, 33)
Long-term follow-up	Necessary but missing	Can long-term lung growth and function be assessed?	Current follow-up frameworks are insufficient	(19–22, 24, 25, 32, 33, 50, 57)

**Figure 2:**
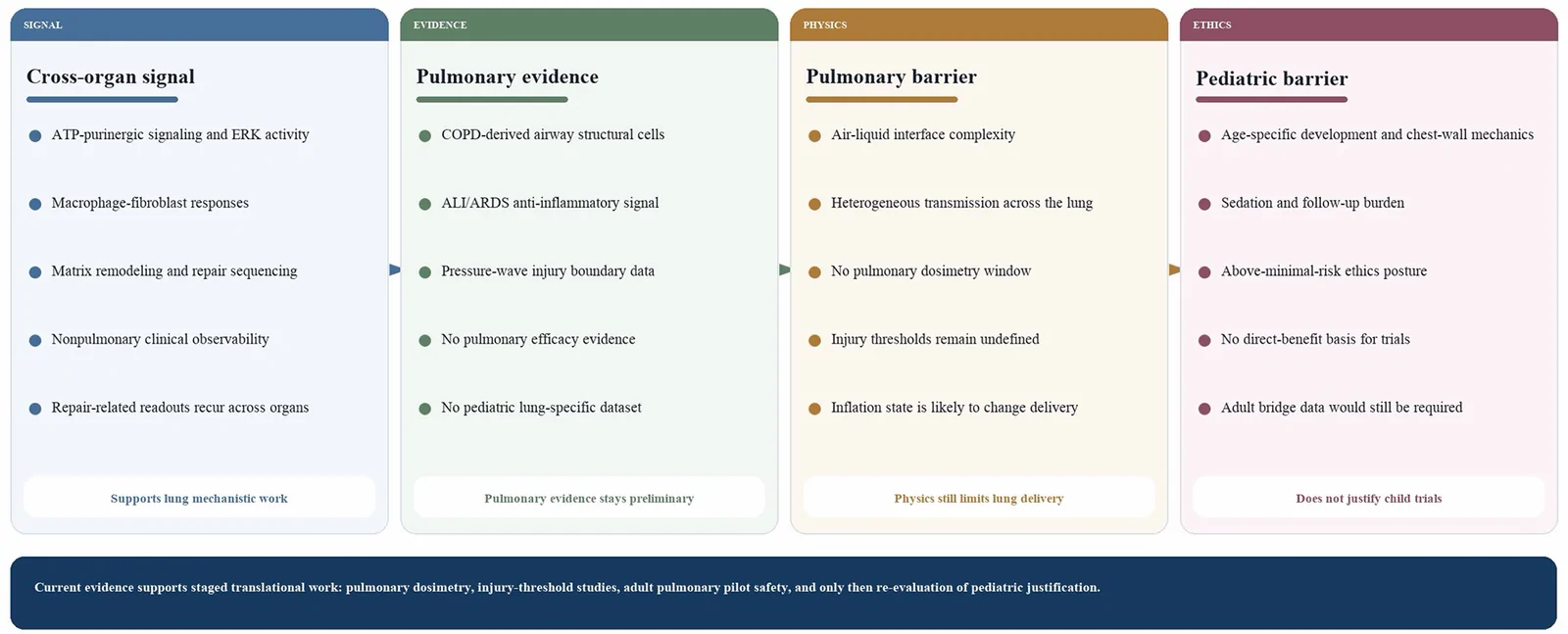
Evidence-to-translation logic for pediatric pulmonary Li-ESWT. Conceptual map linking cross-organ mechanobiology, limited pulmonary preclinical evidence, lung-specific physical barriers, and pediatric ethical constraints. The connecting arrows organize the discussion and do not establish a causal or clinically validated sequence. The figure is intended to show why nonpulmonary Li-ESWT data support biologic plausibility but do not establish pediatric pulmonary efficacy or safety. A more detailed study-level inventory is provided in Supplementary Table S2, and the staged translational sequence is summarized in Supplementary Table S3.

### Sedation and follow-up

Sedation and follow-up are independent sources of risk, not logistical details. Younger children are unlikely to tolerate repeated or technically demanding device sessions without sedation or anesthesia, and cumulative exposure to either the intervention or its anaesthetic context creates a burden qualitatively different from a single adult procedure (35, 62, 67). A meaningful pediatric study would need a prespecified sedation protocol, defined cumulative exposure limits, neurodevelopmental monitoring thresholds, and prospective follow-up covering lung function, growth, rehospitalization, imaging, and quality of life. Designing responsibly around each of these requirements raises the bar for trial entry well beyond what current preclinical evidence can support.

## Discussion

### Feasibility limits

The pulmonary studies establish biological responsiveness in selected experimental systems. The central unresolved issue is whether a response can be reproduced in an intact, aerated lung at an exposure that preserves epithelial and vascular integrity. That question connects the physical delivery problem with the cell-specific mechanisms discussed above.

### Research agenda

Future preclinical studies can connect spatial exposure measurements with epithelial injury, microvascular leakage, inflammatory resolution, and delayed remodeling in the same model (26–28, 30, 43). *Ex vivo* and phantom measurements, followed by small- and large-animal injury-threshold studies, offer complementary ways to address the physical and safety questions. Cell-specific YAP/TAZ activity, *α*v*β*6-TGF-*β* signaling, fibroblast activation, and collagen accumulation would help distinguish organized repair from a persistent profibrotic response (51–58). Developmental injury models additionally require assessment of alveolar and vascular growth, because short-term inflammatory improvement may coexist with disrupted maturation (19, 24, 25, 50, 57, 61). Disease-relevant mechanistic modeling is needed to interpret these responses before adult pulmonary pilot safety work. Supplementary Table S3 summarizes the major unresolved research questions.

Any future studies will also require standardized reporting of wave source, energy flux density, pulse number, treatment frequency, estimated target depth, imaging guidance, sedation strategy, and adverse-event definitions. Without that detail, isolated positive findings will remain impossible to compare, reproduce, or critically evaluate (1, 9, 10, 26–28, 31, 34, 35, 41, 42).

### Overall conclusion

Li-ESWT has measurable mechanobiologic activity in nonpulmonary systems and a limited pulmonary signal from cell and animal experiments (1–8, 26, 27, 29). These findings support lung-directed research on inflammation, stromal responses, and repair. They leave unanswered how local exposure relates to tissue injury and whether early responses predict durable structural benefit.

The key gaps are device-specific lung exposure measurements, pulmonary injury thresholds, adult pulmonary safety data, and developmental risk assessment (19, 24–28, 30–35, 44, 50–57). The evidence reviewed here does not justify pediatric pulmonary clinical trials. Lung-specific dosimetry and preclinical safety characterization, followed by adult safety evidence, remain necessary before pediatric enrollment can be responsibly reconsidered.
