# Supplementary material for: Low-intensity extracorporeal shock wave therapy for pediatric respiratory diseases: current evidence and translational barriers
Source: Front Pediatr. 2026 Oct 27;14:1876906. doi: 10.3389/fped.2026.1876906 (PMC13749039; doi:10.3389/fped.2026.1876906)
Supplement: Supplementary file 1 [file Table1.docx]

Supplementary Material

# Supplementary Methods. Search approach

This manuscript is a narrative review focused on pulmonary Li-ESWT evidence and its relevance to pediatric respiratory disease. Literature selection was PubMed-centered and iterative, supplemented by backward reference screening and targeted consultation of pulmonary mechanobiology, pediatric respiratory biology, research ethics, and device-governance sources. The original search was not designed as a protocol-exhaustive systematic review.

Study selection was guided by pulmonary exposure, biological response, developmental relevance, and safety. Original experiments were prioritized for mechanism and cell or animal findings; systematic reviews were used to contextualize clinical observations in nonpulmonary indications. Studies of lung development, matrix mechanobiology, and pediatric ethics were retained as contextual literature and were not interpreted as Li-ESWT efficacy evidence. Therapeutic cell and animal studies were distinguished from blast-pressure injury studies, and no cross-device dose equivalence was assumed.

During revision, the original publications relevant to pulmonary exposure and mechanosensing were checked again, together with BPD descriptions and reference metadata. The study inventory is selective rather than exhaustive; no screening counts, PRISMA flow diagram, formal risk-of-bias grades, or pooled effect estimates are reported. Tables S1-S3 organize device differences, selected study characteristics, and unresolved research questions.

## Supplementary Table S1. Device and parameter heterogeneity relevant to pulmonary translation

| Dimension | Focused ESWT | Radial or defocused ESWT | Relevance to pulmonary translation | References |
| --- | --- | --- | --- | --- |
| Energy distribution | Can focus deeper in principle | Peak energy remains nearer the contact surface | Pulmonary work needs true energy-mapping, not device labels | (41-43) |
| Focal-depth control | Better suited to deeper targets | Less precise for deeper targets | Chest wall thickness and lesion depth magnify differences | (41-43) |
| Nonpulmonary evidence base | Used across multiple systems | Common in musculoskeletal and superficial tissue settings | Nonpulmonary use does not replace pulmonary dosimetry | (41-42) |
| Extrapolation to an air-filled organ | Unresolved | Unresolved | Device differences do not remove air-liquid interface risk | (28,30,44) |
| Current conclusion | Discussable, not directly translatable | Discussable, not directly translatable | Device-specific studies must precede pulmonary use | (28,30,41-44) |

## Supplementary Table S2. Study characteristics of key cited studies

Note: Exposure settings below are those reported by the original studies, not measured lung-tissue doses. Culture and animal protocols are distinguished where both were used. Settings expressed in bar and mJ/mm² are not interchangeable, and protocols are not intended for cross-study quantitative comparison.

| Study cluster | Organ/model | ESWT type | Protocol detail | Endpoint | Main finding | Supports | Translational gap | Refs |
| --- | --- | --- | --- | --- | --- | --- | --- | --- |
| Direct mechanism | In vitro wound-healing cells | In vitro shock-wave exposure | Original-study protocol; not pulmonary-comparable | ATP efflux, ERK/p38, proliferation, migration | ATP-linked repair signaling | ATP-ERK module | Lung mechanism or clinical dosing | (2) |
| Direct mechanism | Human mesenchymal stem cells | Low-intensity shock-wave exposure | Study-specific | P2X7 activation, osteogenic differentiation | P2X7-linked response | Purinergic mechanotransduction | Pulmonary applicability | (6) |
| Immune response | Chronic wound macrophage model | Low-intensity treatment | Study-specific protocol | Macrophage phenotype and function | Repair-shifted macrophage response | Immune modulation module | Pulmonary delivery or safety | (3) |
| Stromal response | Fibroblast and scar-related models | Low-intensity in vitro treatment | Study-specific; not directly comparable | Fibrosis-related molecules, fibroblast activation | Fibroblast and matrix change | Stromal and matrix readouts | Proven pulmonary antifibrotic efficacy | (4-5,58) |
| Pulmonary cellular response | 16HBE cells; COPD and smoker-control bronchial fibroblasts | Focused piezoelectric ESW; Methods label medium-high energy | Culture: 0.3 mJ/mm², 500 pulses, 4 Hz | Growth-related indices; TGF-β1; procollagen-I | Cell- and time-dependent responses; possible profibrotic effects | Pulmonary cell responsiveness | No intact-lung exposure or calibrated pulmonary dose | (29) |
| Pulmonary animal response | Hyperoxia-induced rat ARDS; rat lung epithelial cells | Focused shock waves; mitochondrial co-treatment groups | Rats: 0.15 mJ/mm², 200 impulses/lung field, 6 Hz, once. Cells: 0.2 mJ/mm², 100 shots | Mitochondrial uptake; lung injury; TGF-β/p-Smad3; collagen | Injury and matrix readouts improved; combined treatment had greater effects | Pulmonary preclinical responses | No in-situ dose measurement or healthy SW-only safety group | (26) |
| Pulmonary animal response | LPS-induced mouse ALI; isolated AT2 cells | Chest treatment; separate culture exposure | Mice: 0.09 mJ/mm², 200 impulses, 10 Hz. Cells: 1.5 bar, 500 impulses, 1 Hz | Mitochondrial and pyroptosis readouts; lung histology | Injury readouts improved; mild abnormalities in healthy exposed lungs | Acute-injury response; short-term safety comparison | No measured parenchymal dose or established injury-free window | (27) |
| Feasibility boundary | Mouse lung pressure-wave injury model | Simulated blast pressure wave, not Li-ESWT | About 337 PSI overpressure; not a therapeutic protocol | Vascular leakage, inflammation, oxidative stress, apoptosis | Lung injury from rapid pressure waves | Feasibility boundary and injury risk | Therapeutic pulmonary safety window | (28) |
| Clinical synthesis | Adult erectile dysfunction trials and syntheses | Multiple low-intensity protocols | Variable across RCTs and syntheses | Functional scores | Short-term effects, limited certainty | Human responsiveness in a mature field | Pulmonary transportability or pediatric relevance | (9,11-12) |
| Clinical synthesis | Chronic wounds and diabetic foot ulcers | Adjunctive low-intensity protocols | Variable; not standardized | Wound-healing outcomes | Adjunctive support in failed repair | Failed-repair concept | Direct lung use | (10) |
| Clinical synthesis | Neurorehabilitation and spasticity | Multiple protocols | Variable across indications and devices | Spasticity and functional outcomes | Functional signal > regeneration claim | Endpoint caution | Pulmonary regeneration claims | (17-18) |

## Supplementary Table S3. Staged translational pathway

| Stage | Core question | Study approach | Ready? |
| --- | --- | --- | --- |
| Stage 1 | How is shock-wave energy distributed across chest wall and lung tissue? | In vitro, ex vivo lung tissue, phantom, and dosimetry studies | No |
| Stage 2 | Can pulmonary injury thresholds, a safety window, and target localization be defined, and disease-relevant responses be interpreted? | Small-animal and large-animal safety studies; disease-relevant mechanistic models | No |
| Stage 3 | Is there acceptable pilot safety in adult pulmonary disease? | Adult exploratory safety and feasibility studies | No |
| Stage 4 | Is pediatric research ethically justifiable? | Only discussable after the first three stages are satisfactorily completed | No |
